# Adverse Childhood Experiences, Substance Use and the Challenges of Help-Seeking among College Students Living in the Midwest

**DOI:** 10.1007/s40653-025-00738-6

**Published:** 2025-09-11

**Authors:** Alexander Wren, Madi DeFrain, Autumn Minor, Rhonda K. Lewis

**Affiliations:** Wichita State University, 1845 N. Fairmount, Box 34, Wichita, KS 67260 USA

**Keywords:** Adverse childhood experiences, College students and perceptions of help-seeking

## Abstract

Adverse childhood experiences (ACEs) are defined as experiences in childhood that increase the likelihood of people engaging in maladaptive behaviors, such as using tobacco or illicit drugs. The current study surveyed 115 undergraduates enrolled at a mid-sized Midwestern University to examine the prevalence of and relationships between adverse childhood experiences and substance use behaviors among college students. A factor analysis was conducted on this college sample to determine how the ACE-10 items were structured. Findings revealed that most students had experienced at least one type of ACE, and that parental divorce was the most ubiquitous form. Results also demonstrated that students who had used a substance at least once in their lifetime experienced more types of ACEs than those who had never used a substance. Limitations and future directions are discussed.

## Introduction

Adverse childhood experiences (ACEs) are defined as a set of highly correlated traumatic life events (e.g., maltreatment, parental divorce, and household dysfunction) that occur before the age of 18 (Anda et al., 1999; Dickie et al., 2024; Felitti & Anda, 2010; Forster et al., 2017a, b). ACEs are alarmingly prevalent in the U.S., with an estimated 38.1% to 55.9% of children having at least one type and 15.0% to 30.6% having two or more (Bethell et al., 2017).

Rates of ACEs are higher when assessed among adults: one study found that over 60% of U.S. adults had at least one ACE and that 25% had three or more (Swedo et al., 2023). ACEs may be particularly common in college populations, with research suggesting approximately 70% of college students had at least one (Forster et al., 2019; King et al., 2024).

Adverse childhood experiences are associated with a range of adverse mental and physical health outcomes in adulthood, including anxiety, depression, disability, and premature death (Dube et al., 2006; Ford et al., 2011; Forster et al., 2019; Karentkin, 2019; Mugoya et al., 2024). The effects of ACEs are transmitted over the life course through cascading impairments in emotional, social, and behavioral functioning onset by disruptions in early neurodevelopment (Giovanelli et al., 2020). To alleviate distress resulting from impaired cognitive functioning and emotional dysregulation, individuals with ACEs often engage in avoidant coping mechanisms (e.g., substance use) that are directly linked to poor adult health (He et al., 2022). Substance use is both common and distinctively high risk as a coping strategy for young adults with adverse childhood experiences (Sebalo et al., 2023). A study that sampled college students in two states found that ACEs were associated with marijuana, tobacco, alcohol, and illicit drug use (Forster et al., 2019). The study, however, did not examine help seeking behaviors among college students nor whether having ACEs affected help seeking in regard to substance use issues.

### ACE Measurement Scales

Following evidence linking early adverse experiences to deleterious health effects in later life, instruments have been designed to assess ACEs in adolescents and adults. Among the most widely used of these tools are various iterations of the ACE Questionnaire. Though widely used, few studies have investigated the psychometric properties of these questionnaires, and fewer still have specifically evaluated the ACE-10: a brief self-report screener of maltreatment and household dysfunction that may be more user-friendly and cost-effective relative to ACE assessments with more complex administration procedures.

Despite the ACE-10's potential utility, uncertainty surrounding its psychometric properties is a substantial drawback to its use for clinical or research purposes. To date, only two known studies have conducted psychometric evaluations of the German (Wingenfeld et al., 2011a, b) and Dutch versions (Van der Feltz-Cornelis & de Beurs, 2023) of the ACE-10 for adults. The ACE-10 demonstrated good reliability and validity in both studies. Van der Feltz-Cornelis and de Beurs (2023) are the only authors to have examined the scale’s factorial structure, finding that a two-factor solution (Factor 1: childhood maltreatment; Factor 2: household dysfunction) had the best fit in Dutch clinical samples. No known studies have thus far evaluated the psychometric properties of the scale’s English version, and the extent to which Van der Feltz-Cornelis and de Beurs (2023) and Wingenfeld et al. and’s (2011a, b) findings generalize remains unclear considering that research has revealed significant cultural heterogeneity in exposure to individual but not cumulative ACEs (Canady et al., 2024). There is thus an extant need for psychometric evaluations of the English version of the ACE-10 conducted in U.S. adult samples. Given the high prevalence of ACEs in the college population, there is a particular need for research that will aid in determining whether the English version is appropriate for use in university healthcare settings. Evidence of this type may be especially beneficial in supporting treatment decision-making by providers working with students who report co-occurring risk factors such substance use issues.

### College Students’ Experiences and Perceptions of Help-Seeking

While research has frequently examined the link between adverse childhood experiences and help-seeking among college students, the literature in this area is generally focused on barriers and facilitators to mental health treatment (Pace et al., 2018). In 2019, Karatekin examined whether ACEs were related to seeking health-related interventions among young adults. The author found that respondents with a greater number of ACEs were more likely to seek both professional and informal help yet simultaneously perceived interventions as less beneficial. Participants in this study most often sought help for mental health problems like depression and anxiety, and the extent to which the findings generalize to help-seeking for substance use reasons remain unclear.

College students often lack awareness of services and perceive stigma in seeking help for health-related concerns (Corrigan, 2004, Yorgason et al., 2008). Such barriers to seeking and receiving help may be particularly relevant to substance use interventions. With the high prevalence of both ACEs (Forster et al., 2019; King et al., 2024) and substance use (Forster et al., 2019) in college populations, there is the opportunity for these to compound additional barriers for help-seeking. While existing literature has explored the impact of both ACEs and substance use on help-seeking behaviors among college students, there remains a need to deepen our understanding of their lived experiences in seeking support.

### Current Study

The overarching goal of this study is to (1) evaluate the psychometric properties of the English version of the ACE-10, as well as (2) investigate the relationship between ACEs and substance use in a sample of college students. This study also aims to contribute to the existing literature by (3) offering insight into where college students who use substances feel comfortable seeking help and (4) the barriers they perceive in accessing that support. By focusing on student voices, this study will add to the understanding of the help-seeking process that college students undertake when seeking help for substance use.

Research questions include:

1. What are the psychometric properties of the ACE-10 in a U.S. sample?
2. What is the relationship between ACEs and substance use among college students?
3. What perceived barriers to and experiences with help-seeking are reported by college students?
4. What resources are needed to increase college students’ willingness to seek help?

## Method

### Participants and Setting

Participants in this study included 115 undergraduate students enrolled at a mid-sized Midwestern University with a total student body of about 15,000. Most participants self-identified as female (73%), with a smaller portion identifying as male (25%) or other/unspecified (2%). Participants were 19 years old on average, with ages ranging from 18 to 50 years. Over half of the sample was white (61%). A smaller percentage of participants were Asian (14%), Hispanic/Latino/Latinx (10%), African American (9%), Native Hawaiian/Pacific Islander (3%), Native/American Indian (1%), or other/prefer not to answer (3%). Detailed demographic information is reported in Table 1.

**Table 1:** Demographics of participants

Gender	N	Percent
Male	29	25%
Female	84	73%
**Age**		
18–20	71	65%
21–24	27	25%
27–31	5	4%
31–50	6	6%
**Race**		
White	79	60.77%
Non-White*	36	39.24%
**Education**		
High school Diploma or GED	21	18.26%
Some College	71	61.7%
2-year degree	10	8.70%
4-year degree	13	11.30%
**Income**		
Less than $25,000	36	31.86%
$25,000-$34,999	11	9.73%
$35,000-$49,000	10	8.85%
$50,000-$74,999	7	6.19%
$75,000-$99,999	9	7.96%
$100,000-$149,999	8	7.08%
$150,000-$199,999	4	3.54%
$200,000 or more	6	5.31%
I prefer not to answer	22	19.47%

^*^The table above shows the demographics of the participants

### Instrumentation

The survey for this study consisted of 80 items, including a demographics questionnaire, open-ended questions related to help-seeking, and the 10-item Adverse Childhood Experiences scale (ACE-10) created by Ford et al. (2014). Sample ACE-10 items include, “Did you have parents or other adults in the household often… punch, grab, slap, or throw something at you?” or “Ever hit you so hard that you had marks or were injured?” and “Was a household member depressed or mentally ill or did a household member attempt suicide?” Substance use history was also assessed using items that asked participants to indicate whether they had ever used tobacco, electronic cigarettes, vapes, alcohol or other drugs. Participants who reported that they had tried one or more of these substances at least once in the past were also asked to indicate whether and how often they had used the substance(s) they endorsed within the past 30 days.

### Procedure

This study was approved by the Institutional Review Board (IRB) at Wichita State University (WSU). The survey was housed in Qualtrics and distributed to WSU undergraduate students via SONA, a departmental research participation platform. Participants were asked to sign a consent form and were required to be at least 18 years old and currently enrolled at WSU to be eligible for participation. The survey took approximately fifteen minutes to complete. To incentivize survey completion, participants were offered entry into a drawing to win a $25.00 gift card.

### Data Analysis

All statistical analyses were conducted in IBM SPSS statistics version 25. Prior to analysis, the data set was cleaned to exclude incomplete cases. Descriptives were first run on demographic variables, including substance use history, and ACE-10 scores. Next, an independent samples t-test and a one-way ANOVA were conducted to assess the relationship between childhood adversity and substance use. The significance level was set as.05 for both tests. Psychometric properties of the ACE-10 were also evaluated. Dimensionality was explored using principal component analysis, while Cronbach’s alpha was used to examine internal consistency.

Thematic analysis was performed by two members of the research team to analyze participants’ open-ended responses. Initial codes were generated then revised as subsets of the data were coded. Once satisfactory interrater reliability was achieved by the two independent raters, themes were developed then refined as needed. Word clouds were later created to visually represent the language of participants’ responses.

## Results

### Quantitative Analysis

#### Descriptives

##### Substance Use

When asked whether they had ever used a substance (i.e., nicotine, marijuana, alcohol, prescription medication, or other), most participants (67%) responded yes. Of those who reported lifetime substance use, approximately half listed alcohol (50.6%) as the first substance they had ever tried. A smaller percentage first used marijuana (16.9%), nicotine (8.4%), prescription pills (2.4%), or other substances (21.7%). Most participants who indicated that they had used a substance at least once in the past also reported currently using one or more substances (62.7%). Alcohol (57.3%) was the most common response to a question asking what substance they had used most recently, followed by nicotine (22.5%) and prescription pills (1.7%). See Table 2 for substance use history.

**Table 2:** Study outcome variables

Variable	*M* or *n*	SD or %
ACE-10		
1. Verbal abuse	.34	.475
2. Physical abuse	.16	.366
3. Sexual abuse	.11	.319
4. Feeling unloved or unsupported	.35	.479
5. Feeling neglected	.07	.257
6. Parental separation or divorce	.42	.496
7. Violence against mother or stepmother	.08	.271
8. Substance abuse by a household member	.28	.451
9. Household member being mentally ill or attempting suicide	.37	.485
10. Household member going to prison	.05	.224
Total score	2.23	2.206
Substance use history		
Currently using	47	41.6%
Having used in the past	28	24.8%
Never used	38	33.6%
First substance used		
Alcohol	42	50.6%
Marijuana	14	16.9%
Nicotine	7	8.4%
Prescription pills	2	2.4%
Other	18	21.7%
Last substance used		
Alcohol	51	57.3%
Nicotine	20	22.5%
Prescription pills	2	2.2%
Prefer not to say	16	18.0%

##### Adverse Childhood Experiences

Table 2 outlines participants’ ACE-10 scores. Though some participants in the current study had never experienced any of the events measured by the ACE-10, most had experienced at least one type (72.3%). Only 7.1% of the sample had experienced six or more types, with the majority reporting between one and five (*M* = 2.23). Participants most frequently experienced parental divorce (42.0%), a household member having mental illness or attempting suicide (36.8%) and feeling unloved or unsupported by family members (35.1%). Fewer participants reported verbal abuse (33.9%), a household member abusing substances (28.1%), or physical (15.8%) or sexual abuse (11.4%). A minority witnessed violence against their mother or stepmother (7.8%), experienced neglect (7.0%), or had a household member go to prison (5.3%).

#### ACE-10 Psychometric Properties

An EFA using principal component analysis was conducted to explore the factor structure of the ACE-10. Bartlett’s test of sphericity was significant (χ^2^ (45) = 204.76, *p* <.001), indicating the correlation matrix was not random. The Kaiser–Meyer–Olkin measure of sampling adequacy was acceptable (.745). The correlation matrix was subsequently deemed appropriate for factor analysis.

Velicer’s minimum average partial test and a scree plot of the eigenvalues were used to determine the appropriate number of factors to retain. Both indicated a one factor solution. When a one factor solution was enforced, nine of the ten items loaded saliently onto the factor. The only item that failed to demonstrate a significant loading was a measure of childhood experiences of sexual abuse. The EFA was conducted again, excluding the item that failed to load, and the resulting solution accounted for 33.4% of the variance. These findings suggest that the ACE-10 should be interpreted as a unidimensional construct. Factor loadings are presented in Table 3.

**Table 3:** Factor loadings for ACE-10 items

Items	Factor 1 Loading
Verbal abuse	.570
Physical abuse	.459
Feeling unloved or unsupported	.602
Feeling neglected	.609
Parental separation or divorce	.610
Violence against mother or stepmother	.493
Substance abuse by a household member	.634
Household member being mentally ill or attempting suicide	.599
Household member going to prison	.599

For a scale to be considered reliable, Cronbach’s alpha must exceed 0.70. Cronbach’s alpha for the ACE-10 was calculated as .763 prior to item deletion. The alpha value increased to .766 after the item that failed to load was removed. Both values fall within the acceptable range of internal consistency.

#### Relationship between ACEs and Substance Use

An independent samples t-test was conducted to compare ACE-10 scores between individuals who had and had not used a substance (i.e., nicotine, marijuana, alcohol, prescription pills, or other) at least once in their lifetime. Individuals who had used a substance at least once (*M* = 2.61, *SD* = 2.30) reported significantly higher ACE scores than those who had never used a substance (*M* = 1.46, *SD* = 1.80; *t*(110) = −2.68, *p* =.022). The magnitude of the mean differences was moderate (*d* = 0.56).

A one-way ANOVA was conducted to compare ACE-10 scores between individuals who had never used a substance (i.e., nicotine, marijuana, alcohol, prescription pills, or other), individuals had used a substance at least once in the past, and individuals who were current users of one or more substances. There was a significant main effect at the *p* <.05 level for the three groups [*F*(2, 108) = 5.12, *p* =.007]. The effect size was moderate to large (η^2^=.087). Post-hoc comparisons using Tukey’s HSD revealed that the mean score for current substance users (*M* = 2.96, *SD* = 2.44) was significantly higher than that of individuals who had never used a substance (*M* = 1.46, *SD* = 1.80, *p* =.006). No significant differences in mean scores were found between the other groups. 

### Qualitative Analysis

#### Influence of Substance Use

**Fig. 1:**
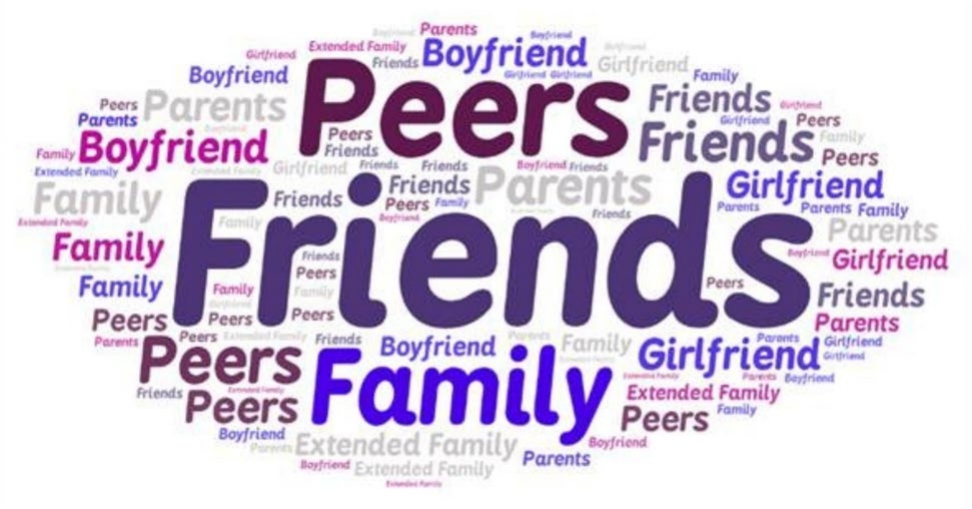
Word cloud of responses to who introduced you to drugs?

**Fig. 2:**
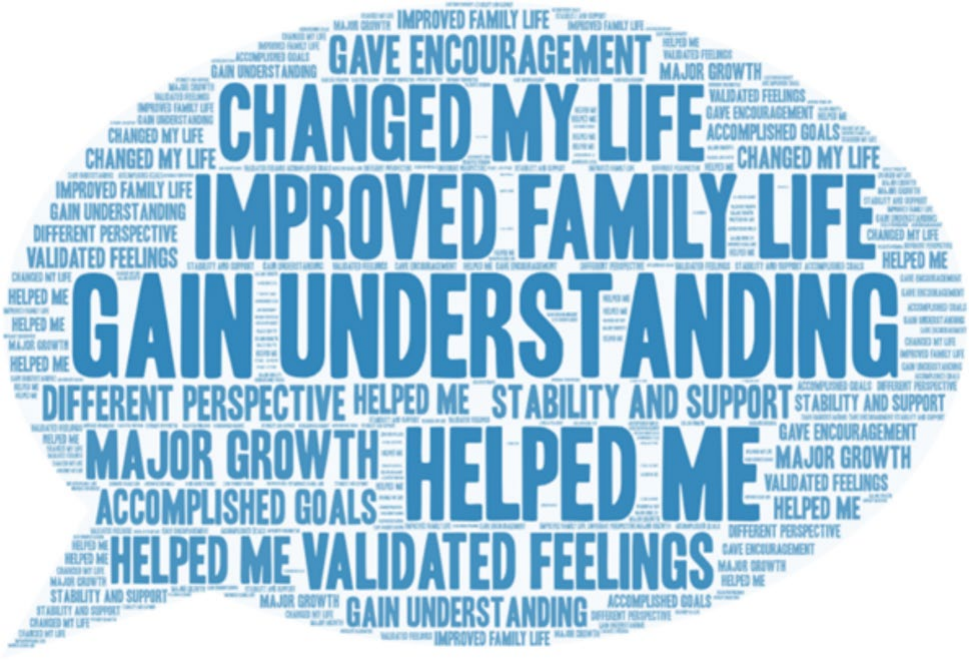
Word cloud of responses to How counseling was helpful?

Table 4 captures participants'open-ended responses to questions about the influence of substance use on their lives. One participant stated, “My dad used drugs frequently and I wanted a better life for myself.” Others similarly reported wanting to take a different path after seeing family members struggle with substance use. “Seeing my father’s and my sister’s struggle with alcohol abuse it has made me avoid drinking if I am upset/stressed and I do not drink often I know it can be a dangerous path.” See Fig. 1, 2, 3 for participant’s responses to open-ended questions about influences related to substance use and help-seeking.

**Table 4:** Participant’s open-ended responses to questions about help-seeking

Question	Major Theme(s) Subthemes	Quotes
Yes-Do you feel that substance abuse/misuse or the exposure of substance abuse/misuse has influenced your decision to pursue college education?	Influence of substance use Subtheme: Desire for Better Life	“Somewhat. I pursued a college education to give my kids a better life, and it's ultimately all about them, but I think some of that stems from wanting to give them a better childhood than I had. And drugs and alcohol and mental illness had a large impact on the abuse that I suffered as a child and teenager.”; “My dad used drugs frequently and I wanted a better life for myself.” “seeing someone close to you choose a path of substance abuse and ruin their life makes you want to never be that person and be better and have a better life than that.”
Was there outside influence that led to your substance abuse/misuse? (Elders, Peers, Family friends, Etc.) Please be specific. * Please note that no personal information is needed such as names. All that is needed is the relationship/or how you know the person. EX: Best friend, Mothers friend…	Friends and Peers	“I have only drank like 5 times in my life, but the first time I did it was with a small group of close friends, and they made sure that I didn't drink more than I should've. So far, I'm fairly sure I'm not addicted”
Did substance abuse/misuse have any influence on you to take a different path and lean toward sobriety? (Even if it was others whose substance abuse/misuse that led you to this conclusion)	Family Use influenced different path	“Seeing my father's and my sister's struggle with alcohol abuse it has made me avoid drinking if I am upset/stressed and I do not drink often because I know it can be a dangerous path.”; “I have an addictive personality and seeing how it destroyed members of my family and influenced the way I was treated has prevented me from doing anything illegal. When I had my children and surgery, I did not even take pain meds beyond standard ibuprofen for this reason.”; “My brother is a hard-core drug addict, and he has almost died from drugs. I've also seen too many people be negatively affected by drugs.”
If you did have counseling, was it helpful Do you feel that the counselor was efficient in helping you progress?	Counseling was helpful Subthemes: Helped Gain Understanding Support, Validation, Growth	“I did find it helpful even though I was reluctant at the beginning. I found tips and tricks to help myself to quit on my own.” “Yes, my therapist was the first person who attuned with my feelings and validated my trauma.” “Still with them to this day, but major personal growth over the last year.”
Were there any outside influences that may have compromised your counseling in any way such as: Job, Peers, Substances, Family, etc	Factors that compromised your counseling Subtheme: Influences by peers and family, Cost of therapy	“I was not in school because I was unable to fill out the FAFSA due to my parents'violence and kicking me out, so I was really depressed. I felt powerless in my situation, and I felt that my life had no purpose.” “Therapy is expensive.”
How do you think psychologists can better their practices to help prevent substance abuse/misuse? Do you think they can?	Counseling Practices be improved? Subtheme: Construct more individualized treatment	“Focus on prevention. Find the root cause of the addiction tendencies and provide resources, support systems around the clock, 24/7 for individuals like me. Let them know that the cravings don't make them bad people. Help them strengthen themselves so they can fight the cravings and trauma memories more easily,” “Yes, psychologists can better their practices by going to different classes and obtain different certificates to better their knowledge of substance abuse/misuse. It takes a while for people to find the right type or form of therapy. If they are better equipping for the situation given, then they could also continue sessions with that client.”
Is there another source that you might recommend seeking help from besides a counselor?	Strong family connections Subthemes: Structures Community Support Rehab Support groups Family	“Besides a counselor, someone struggling with substance abuse/misuse needs to have social support through friends and family that will not negatively affect their progress through sobriety.” “Something that is available all times of the day. I struggle most at night where I can't get ahold of anyone and that's when I feel most alone and vulnerable.” “​​I toured a rehab facility or two, debating on whether or not to go to one. First off, I could not afford one and at the time I had no idea what narcotics anonymous. I found hearing their methods of how they were planning on helping you get off the substance as good little tools to help myself do it on my own.”

**Fig. 3:**
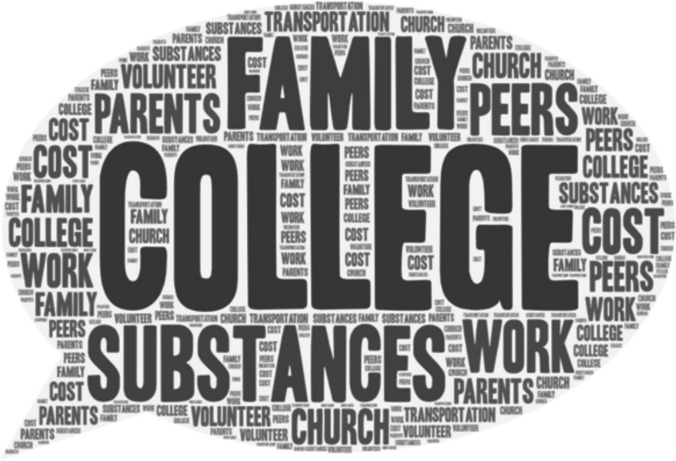
Word cloud of responses to factors that compromise your counseling?

#### In What Ways can Counseling Practices be Improved for Substance Abuse and Misuse?

One respondent stated that providers could “focus on prevention.” Another respondent suggested “[providing] resources, support systems around the clock, 24/7 for individuals like me. Let them know that cravings don’t make them bad people. Help them strengthen themselves so they can fight the cravings and trauma memories more easily.”

#### Recommendations for Substance Use/Misuse Treatment

Participants had a number of recommendations. One participant recommended, “Besides a counselor, someone struggling with substance abuse/misuse needs to have social support through friends and family that will not negatively affect their progress through sobriety.” Another participant suggested a facility that might be available all during the day for example. “Something that is available all times of the day. I struggle most at night where I can’t get a hold of anyone and that’s when I feel most alone and vulnerable.”

## Discussion

The current study explored the relationships between adverse childhood experiences, substance use, and the challenges of help-seeking among college students. The findings showed that participants in this study experienced ACEs at similar rates (72%) to those in other studies (70%; Forster et al., 2019). Participants also engaged in substance use at similar rates to other college samples (American College Health Association, 2019). This study additionally examined help-seeking perceptions and experiences. We found that a greater portion of this sample reported seeking counseling (52%) than the percentage reported in national samples (28.9%; Xiao et al., 2017). Although participants in this study stated they sought help, 40% described their counselor as not being helpful and listed a variety of factors that compromised their investment in counseling, such as work-related conflicts and transportation.

The first aim of this study was to evaluate the psychometric properties of the ACE-10. To our knowledge, this is the first known study to evaluate the dimensionality of the English version ACE-10, using a sample of U.S. college students. Our results affirmed that the questionnaire has a unidimensional structure, but the one factor solution was found to support only nine of the 10 items. The item used to measure sexual abuse did not load, contrasting with Van der Feltz-Cornelis and de Beur’s (2023) finding that all 10 items load saliently onto factors of household dysfunction and childhood maltreatment. This discrepancy may be attributed to cultural differences reflected between the current study’s sample of U.S. college students and Van der Feltz-Cornelis and de Beur’s (2023) sample of Dutch out-patients, as research has evidenced variation in the types, severity, and prevalence of ACEs across country contexts (Alcaraz et al., 2024).

The second aim of the study examined the relationship between substance use and ACE-10 scores. The study found a statistically significant association between ACE-10 scores and substance use history, with those who had used a substance at least once reporting more ACEs than those who had never used a substance. More specifically, the average score of current (but not prior) users was higher than that of individuals who had never used a substance. These findings expand empirical support for the existence of a relationship between ACEs and substance use in adolescence and early adulthood (Dube et al., 2003). Taken together, evidence suggests that screening adolescent populations for ACEs may be an effective prevention strategy as this practice could create targeted interventions for individuals identified as at-risk. In addition, given the current mental health, and stress that college students are experiencing getting a better understanding of the backgrounds that college students have will create a trauma informed campus that can meet the needs of students and provide the resources they need as they enter college. This was stated in the comments from participants of having 24-hour resources when someone may need to talk to a professional in the middle of the night.

The third aim of the study was to examine what resources were needed to increase college students’ willingness to seek help. The results showed that this sample of college students were seeking counseling; approximately 12% found it helpful and 40% stated counseling was not helpful and 48% preferred not to answer. Thus, highlighting access to help but the quality of the help-seeking encounter may need to improve. However, for those who did find it helpful participants reported being able to set goals and have a stable voice and support. Thus, it is key for counselors and client matches to be made so that students, especially on college campus, can receive access to the proper resources they need. Imbedded in this aim was the ways that outside influences may have compromised counseling for students. Students mentioned their job, family and paying for therapy being expensive as a barrier for treatment. The cost of treatment for example was a barrier that can be addressed by universities and colleges. For example, universities and colleges might offer services on a sliding scale and low-cost options to students.

The final aim was to examine recommendations for seeking help. Participants made a number of recommendations that would help universities to have help lines available 24 hours when people are having crisis. For instance, the new 988 suicide help line is available and has crisis counselors around the clock. Universities have deployed mental health apps (Lattie et al., 2022) or telehealth professionals are available to reduce stigma and help make mental health and substance abuse treatment more accessible (Ollio et al., 2022).

### Strengths and Limitations

The practical importance of the current findings constitutes a major strength of this study, as it is the first to examine the psychometric properties of the ACE-10 in a U.S. adult sample. Previous psychometric evaluations of the scale were limited to studies assessing the Hungarian version for adolescents (Kovács-Tóth et al., 2023) and the Dutch (Feltz-Cornelis & Beurs, 2023) and German versions for adults (Wingenfeld et al., 2011a, b). As a result of this study, the English version of the ACE-10 item survey has now been validated in the United States as a reliable measure of childhood maltreatment and household dysfunction.

Secondly, the results focused on a mixed methods approach using the survey results of participants and the lived experiences of participants in their own words to describe their thoughts. These results allowed us to gather data about whether counseling was helpful and if anything compromised gaining access to counseling services. Thus, the mixed methods approach allowed us to examine the ACE-10 as a tool, the relationship between ACEs and substance use, help seeking among college students and how colleges can use the ACE-10 as a screening tool to create programs for college students and tailor their programs to adjust to the challenges that college students are faced with (transportation, limited insurance) and aspects of quality in the counseling experience. Limitations are also noted. Firstly, this study did not include a validated help-seeking measure. Although participants were asked to describe any factors that may have compromised them getting help, no scale was developed to assess specific help-seeking behaviors. For the items that asked about access to and perceptions of counseling services, it was unclear whether participants’ responses related to substance use interventions or mental health care in general. The inclusion of a comprehensive help-seeking measure might have assisted in correlating behavioral factors, which may have informed the development of future interventions. Secondly, this study also used a convenience sample, which might not accurately represent the racial and ethnic diversity of the larger university population. Thirdly, this sample was heavily female and may not reflect the diversity in terms of gender identity, although the campus community is predominately female.

Lastly, it is important to gather information about those experiencing trauma along with the ACE-10 (e.g., death of a parent) for college students and their willingness to seek and receive help. There is also a need in the future to conduct a more in-depth analysis of the drug using history of current users. A number of participants reported using substances but stated their substance use was not a problem, and that it did not interfere with school. It would be important to examine how participants viewed substance use and how this might impact help-seeking behavior in the future.

## Conclusion

Our research provides an overview of the connection between ACE-10 scores and substance use and the complexity of help-seeking perceptions of college students. In our study college students sought counseling and stated that the services were not always helpful or available when they needed services the most. For colleges and universities to effectively intervene we recommend colleges and universities to systematically assess their students to determine their individual risk levels and provide tailored help-seeking options. Higher educational institutions might consider expanding their advertising of free or reduced-price services like counseling to make students more aware of their existence. Community-based support is critical in recovery from both substance abuse and trauma, including meditation rooms in campus buildings, hotlines available late at night when crises occur, and programs designed to limit access to intoxicating substances. In addition, universities can implement quality control practices to improve counseling experiences for college students.

## Data Availability

NA.
